# Utility of endoscopic neodymium-iron-boron magnet ring tracer technique in laparoscopy for colorectal lesions

**DOI:** 10.1055/a-2499-7462

**Published:** 2024-12-19

**Authors:** Song Yang, Qian Du, Hongling Li, Weiping Liu, Rui Xie

**Affiliations:** 1 56663 Endoscopy and Digestive System, Guizhou Provincial Peopleʼs Hospital, Guiyang, China

With the rapid development of endoscopic diagnostic and therapeutic techniques, endoscopic mucosal resection (EMR) and endoscopic submucosal dissection (ESD) have become the main surgical procedures for the treatment of early-stage colorectal cancer. However, for small lesions infiltrating deeper than SM1 (SM1 refers to the upper third of the submucosal layer), tumors that have not yet penetrated the serosal layer, or lesions with positive margins after endoscopic treatment that require additional surgical intervention, precise lesion localization during laparoscopic surgery poses a significant challenge 1 . This difficulty arises because the lesion cannot be palpated by conventional means, making it difficult to achieve precise resection within a small area and potentially leading to incorrect resection of bowel segments or missed lesions. Traditionally, localization during laparoscopic surgery has relied on the injection of staining agents such as nano-carbon or indigo carmine around the submucosa of the lesion. However, in clinical practice, these staining agents often disperse, leak, or become difficult to identify after prolonged injection, highlighting the need for a safe, efficient, and precise method to localize colorectal tumor lesions 2 3 4 5 . We have adopted a neodymium-iron-boron (NdFeB) magnetic ring marking method that utilizes magnetic attraction to rapidly achieve intraoperative localization with an accurate localization rate of 100%.

A 45-year-old male patient presented with “unformed stools for 1+ years” and was referred for further evaluation. Colonoscopy revealed an adenoma in the transverse colon, with endoscopic evaluation indicating that the lesion was locally infiltrating into the deep submucosa or the underlying muscular layer. Computed tomography (CT) imaging suggested significant thickening of the transverse colon.

Materials used included: NdFeB magnetic rings (nickel–copper–nickel 3-layer coating for rust and corrosion resistance, 8 mm in diameter, 3 mm in thickness), rotatable reusable soft tissue clips (harmony clip; Nanwei Medical), a CKLV-260SL main unit, and a CJ-Q260 colonoscope (Olympus, Japan).

Magnetic ring placement was performed by a dedicated physician in all patients. First, surgical suture thread was passed through the magnetic ring and knotted ( Fig. 1 ). A harmony clip was then passed through the biopsy channel of the colonoscope and opened. The other end of the suture was tied to one of the clip arms, and the clip was closed and retracted into the biopsy channel, positioning the magnetic ring at the side of the tip of the colonoscope ( Fig. 2 ). The colonoscope was inserted through the anus, and the magnetic ring–harmony clip device was advanced to the lesion site. The magnetic ring was then fixed at the lesion site or within 0.5 cm of the lesion using two harmony clips ( Fig. 3 ). After placement of the magnetic ring, patients were advised to avoid strenuous exercise and to stay away from high magnetic field environments, such as MRI rooms, until the magnetic ring was removed.

**Fig. 1:**
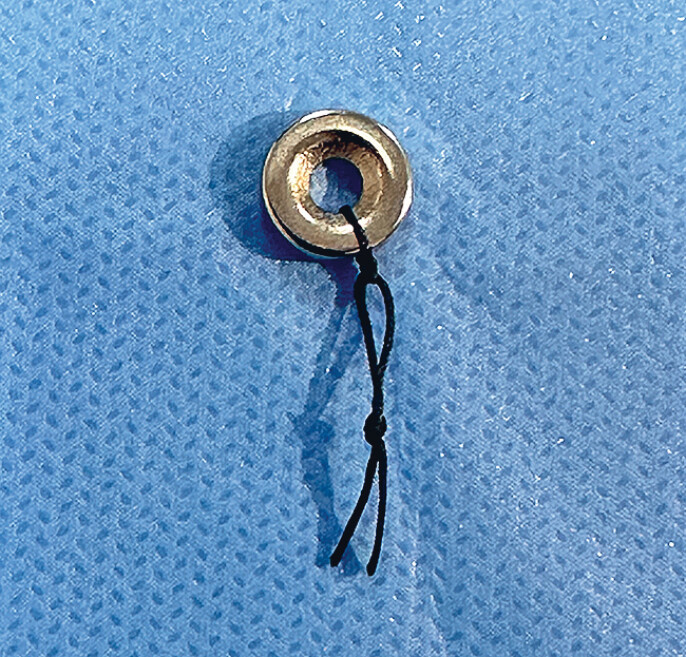
Surgical suture thread passed through a neodymium-iron-boron (NdFeB) magnetic ring and knotted.

**Fig. 2:**
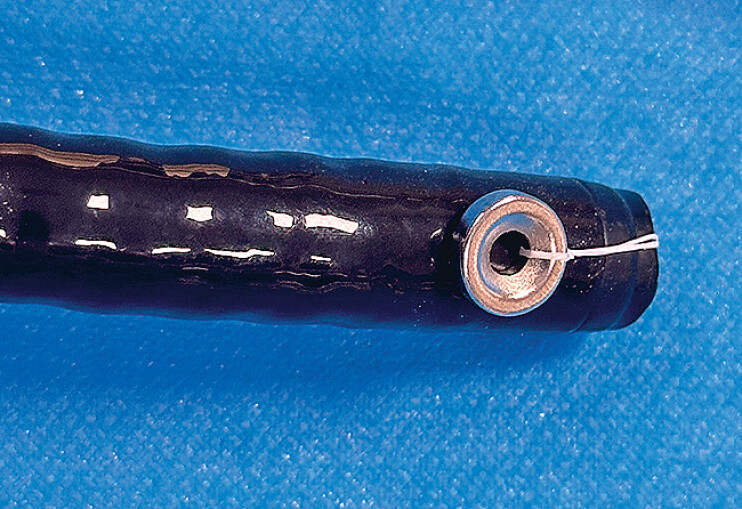
The marking magnetic ring is positioned at the side of the colonoscope tip.

**Fig. 3:**
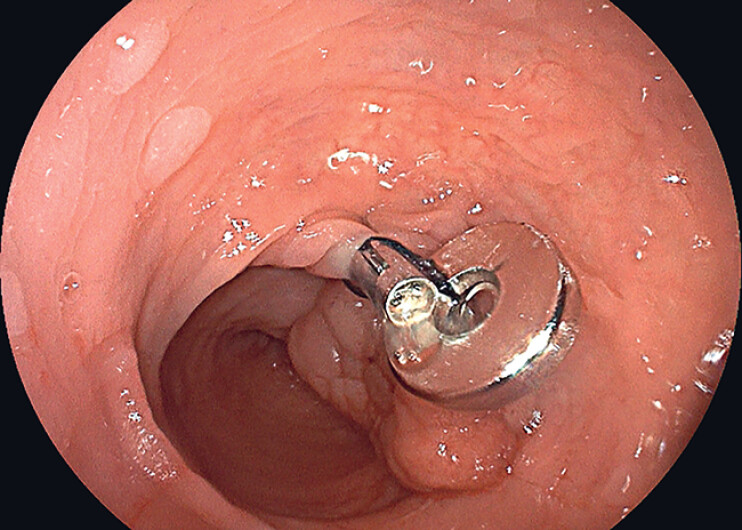
Two harmony clips secure the magnetic ring at the lesion site.

All patients underwent laparoscopic radical surgery on the day after magnetic ring placement. Depending on the location of the lesion, trocars were placed in the abdomen. The affected bowel segment was mobilized under laparoscopic guidance. Another magnetic ring, sterilized with ethylene oxide and tied with surgical sutures, was introduced into the abdomen through a laparoscopic trocar using surgical forceps. The previously placed magnetic ring was then located, and as the forceps approached the marked site, the two magnetic rings were visually observed to attract each other and rapidly converge through the bowel wall. This allowed accurate intraoperative localization of the lesion ( Fig. 4 ). The extent of the intestinal segment to be resected was determined based on the characteristics of the lesion. At the end of the procedure, the magnetic ring–harmony clip device was removed from the body along with the specimen via laparoscopy ( Fig. 5 ).

**Fig. 4:**
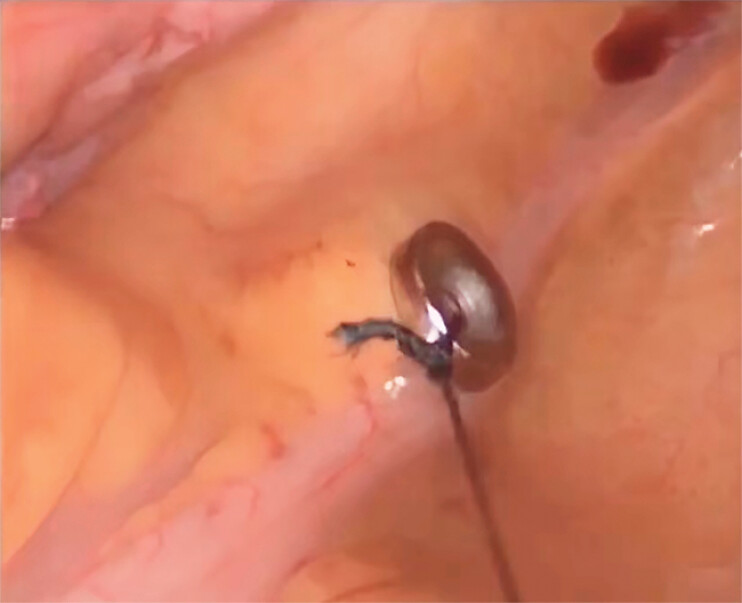
The locating magnetic ring and the marking magnetic ring attract each other through the intestinal wall, pinpointing the lesion site.

**Fig. 5:**
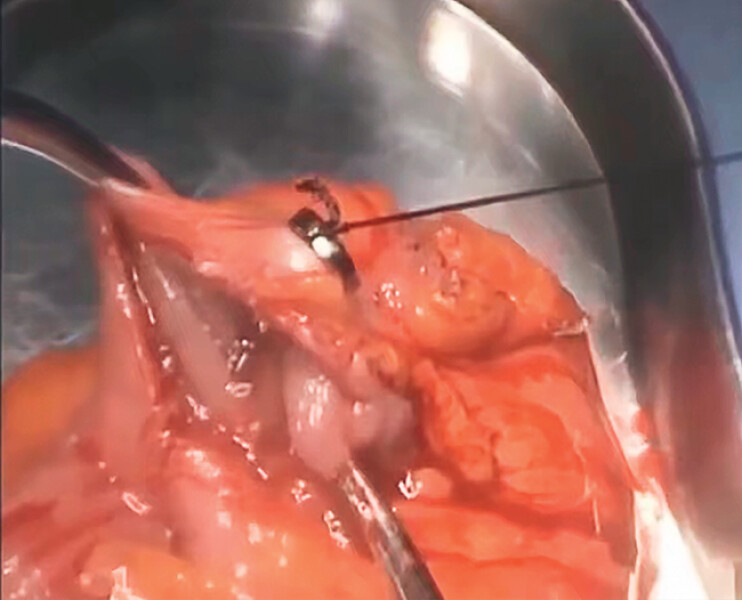
The resected intestinal segment is removed along with the magnetic ring–harmony clip device.The tumor lesion is exposed by opening the intestinal wall next to the locating magnetic ring.

Preoperative marking of the lesion with an NdFeB magnetic ring to aid laparoscopic localization enhances the safety and precision of laparoscopic surgery. This technique is simple, convenient, inexpensive, and allows rapid and accurate localization, making it a valuable method for clinical use and worthy of broader application ( Video 1 ).

The neodymium-iron-boron (NdFeB) magnetic ring marking method is used to quickly and accurately locate a lesion under laparoscopy, using the principle of magnetic attraction.Video 1

Endoscopy_UCTN_Code_TTT_1AT_2AZ

## Notes

Endoscopy E-Videos https://eref.thieme.de/e-videos
*E-Videos* is an open access online section of the journal *Endoscopy* , reporting on interesting cases and new techniques in gastroenterological endoscopy. All papers include a high-quality video and are published with a Creative Commons CC-BY license. Endoscopy E-Videos qualify for HINARI discounts and waivers and eligibility is automatically checked during the submission process. We grant 100% waivers to articles whose corresponding authors are based in Group A countries and 50% waivers to those who are based in Group B countries as classified by Research4Life (see: https://www.research4life.org/access/eligibility/ ). This section has its own submission website at https://mc.manuscriptcentral.com/e-videos .
